# Efficient gene transfection to lung cancer cells via Folate-PEI-Sorbitol gene transporter

**DOI:** 10.1371/journal.pone.0266181

**Published:** 2022-05-04

**Authors:** Kye Soo Cho, Sanghwa Kim, Hyung Bin Chun, Jae Hee Cheon, Myung-Haing Cho, Ah Young Lee, Rohidas B. Arote

**Affiliations:** 1 Department of Tumor Immunology, National Cancer Center, Goyang, Republic of Korea; 2 Department of Internal Medicine, Yonsei University College of Medical Science, Seoul, Republic of Korea; 3 Laboratory of Toxicology, Research Institute for Veterinary Science and College of Veterinary Medicine, Seoul National University, Seoul, Republic of Korea; 4 Department of Life Science, Waterloo University, Waterloo, Ontario, Canada; 5 RNABIO, Seongnam, Gyeonggi-do, Republic of Korea; 6 Department of Molecular Genetics & Dental Research Institute, School of Dentistry, Seoul National University, Seoul, Republic of Korea; Central University of Rajasthan, INDIA

## Abstract

Lung cancer is known to be one of the fatal diseases in the world and is experiencing treatment difficulties. Many treatments have been discovered and implemented, but death rate of patients with lung cancer continues to remain high. Current treatments for cancer such as chemotherapy, immunotherapy, and radiotherapy have shown considerable results, yet they are accompanied by side effects. One effective method for reducing the cytotoxicity of these treatments is via the use of a nanoparticle-mediated siRNA delivery strategy with selective silencing effects and non-viral vectors. In this study, a folate (FA) moiety ligand-conjugated poly(sorbitol-co-PEI)-based gene transporter was designed by combining low-molecular weight polyethyleneimine (LMW PEI) and D-sorbitol with FA to form FPS. Since folate receptors are commonly overexpressed in various cancer cells, folate-conjugated nanoparticles may be more effectively delivered to selective cancer cells. Additionally, siOPA1 was used to induce apoptosis through mitochondrial fusion. The OPA1 protein stability level is important for maintaining normal mitochondrial cristae structure and function, conserving the inner membrane structure, and protecting cells from apoptosis. Consequently, when FPS/siOPA1 was used for lung cancer *in-vitro* and *in-vivo*, it improved cell viability and cellular uptake.

## Introduction

Gene therapy has received much attention as an alternative to cancer treatment. Lung cancer is in the center of this attention, considering that it is the primary cause of death in America and According to the SEER Cancer Statistics Review(CSR), the survival rate is still less than 15–20% [1]. As an ideal alternative to gene therapy, it must be able to efficiently and safely deliver nano-complexes to target cells. Successful delivery of the nano-complex depends on the carrier and its transfection efficiency. Typical major carriers include: viral vectors `/ non-viral vectors [2,3]. Viral vectors have been reported to have high transfection efficiencies, but their characteristics, such as size limitation, risk of immune response, and associated toxicities, limit therapeutic aim and effect. Therefore, non-viral carriers are preferred to viral carriers [2].

Among non-viral gene carriers, polyethyleneimine (PEI) has been widely used as the standard due to its advantages, including complex formation with genes and rapid endosomal escape via the proton sponge effect. However, PEI is barely suitable as a carrier for gene therapy because of limitations such as high toxicity [4]. To overcome this obstacle, the gene carrier has to be chemically modified to enhance biocompatibility and reduce toxicity. Our group has previously developed a variety of PEI-mediated co-polymers, including poly(sorbitol-co-PEI), PSMT, polymannitol, polyglycerol, and polylactitol [5,6].

In recent years, receptor targeting using folate as the ligand has emerged as the novel target moiety for cancer therapy. Folate receptors (FR) are highly expressed in cells of different cancer types. Additionally, folate binds strongly to FR (Kd 0.01–1 nM) in lung cancer cells as well as other types of c-ancer cells [7].

Folate is also preferable for cancer therapy because it is non-immunogenic, it easily conjugates with other polymers, and it is inexpensive. Therefore, the folate-conjugated targeting strategy is promising in the cancer therapy field. Combining folate with a cancer-targeted gene delivery system, such as folate-conjugated PSMT (FPS), could enhance the efficacy of ligand targeting and efficiently deliver the nano-complex to the cancer cells in a greater proportion relative to normal cells.

SiOPA1 was used to target OPA1, one of the most significant mitochondrial proteins. OPA1 plays a key role in regulating the mitochondrial fusion process [8]. Additionally, degradation of OPA1 leads to the apoptosis of mitochondria, which also has an effect on cancer [9]. In this study, siOPA1 was loaded into PSMT (conjugated with folic acid) and tested both *in-vitro* and *in-vivo* to determine the efficacy of the siOPA1/FPS polymer as a lung cancer treatment application.

The nanoparticle/siRNA complex is a promising new approach, due to its selective silencing effect on oncogenes and the effectiveness of a multi-functional nano-carrier for gene delivery. This complex also resonates with new trends in the field of cancer therapy.

## Materials and methods

### Ethics statement

The mice were placed in a nose-only exposure chamber, and 30 ml of the FPS/siRNA complexes was inhaled via aerosol delivery using a nebulizer for 30 min, twice per week for 4 weeks, measured the number and size of tumors, sacrificed using Co2 gas All experimental protocols were reviewed and approved by the Institutional Animal Care and Use Committee (IACUC) of Seoul National University(SNU-160615-6) and abide by the Institute of Laboratory Animal Resources (ILAR) guide.

### Reagents

Branched PEI 1.2kDa, Folate, sorbitol diacrylate (SDA), dimethyl sulfoxide(DMSO), dicyclohexylcarbodiimide (DCC), N-hydroxylsuccinimide(NHS), Acryloyl chloride Dimethylformamide(DMF), were purchased from sigma-Aldrich (St.Louis, Mo, USA), tetrazolium reagent(MTT), bafilomycin A1, chlropromazine(CH), genistein(GE), wortmannin, were ordered from Sigma Aldrich (St. Louis, Mo, USA), Roswell Park Memorial Institute(RPMI), Fetal bovine sereum(FBS), Dulbecco’s modified Eagle’s medium(DMEM) and penicillin /streptomycin were purchased from Hyclone (Logan, Utah) for *in-vitro*. 0.1U RNase A was purchased from sigma-Aldrich (St.Louis, Mo, USA), siOPA1 (siRNA) was purchased from Bioneer company(Dae-jeon, South of Korea), pGL3 gene for control vector, plasmid maxiprep kit for E.coli plasmid purification and luciferase assay system for *in- vitro* transfection study were purchased from Promega (Madison, WI, USA).

### Synthesis of poly(sorbitol) and polysorbitol-based gene transporter

Sorbitol diacrylate (SDA) was prepared by reaction between sorbitol and acryl chloride for linkage with amine group of PEI(M.W 1.2kDa) according to our previous paper [10]. Folate(FA) was diluted in 6ml of DMSO with 9.5mg(0.046mmol) of DCC added to the solution. After completion of synthesis, polymer was dialyzed in DW using Spectra/PorR membrane [molecular weight cut off (MWCO): 3500Da; Spectrum Medical Industries, Inc, LosAngeles, CA, USA] at 4°C for 24H. Dialyzed FA/PSMT (FPS) was freeze-dried until the polymerization was completed. Then FPS polymer was stored at -70°C until next scheduled experiments. FPS was characterized by using ^1^H NMR spectroscopy (AVANCE 600, Bruker, Germany) for confirming the composition of the FA in PSMT.

### Physicochemical characterization of FPS

FPS was characterized using a 1H NMR spectrometer (AVANCE 600, Burket -600mHz, Germany) for estimating the composition of FPS in PSMT. The molecular wight of FPS was confirmed using a gel-permeation chromatography column (GPC) with TSKgel G5000PWxl-TSKgel and G3000PWxl-C in fixed 45°C with a flow rate of 1.0 ml/min and 0.1M NaNO_3_ was used as the mobile phase. Particle size and zeta-potential of FPS were measured using a dynamic light scattering spectrophotometer (DLS) (Otsuka Electronics DLS-7000). FPS/siRNA complexes were made at N/P ratio 5,10,15 and 20 with 50pmole luciferase siRNA in 2ml of total volume for measuring the nanoparticle sizes. The complexes were prepared with double distilled water (DDW) on a copper grid.

After drying the copper grids, the morphology of the FPS/siRNA(siOPA1) complexes were observed at N/P ratio 20 by energy-filtering transmission electron microscopy (EF-TEM) (LIBRA 120, Carl Zeiss, Germany).

### Measurement of osmolarity

To measure osmolarity, 50μL of samples including bPEI 1.2K, PSMT and FPS in DW with various concentration (mg/ml) was prepared. Measurement was calculated by using cryoscopic osmometer (Osmomat 010, Gallay Medical & Scientific, Australia)

### Cell lines

Cell lines (A549, HepG2, 293T cells) were purchased from Korea Cell line bank (ATCC), A549 and HepG2 cells were cultured in RPMI-1640(Thermo Fisher Scientific, Waltham, MA), and 293T cells were cultured in DMEM(Thermo Fisher Scientific, Waltham, MA). All cell lines contained FBS 10% and Penicillin, and were maintained at 37°C in a 5% CO_2_ humidified incubator.

### Animals

K-ras^LA1^ lung cancer model mice were purchased from the Human Cancer Consortium-National Cancer Institute (Frederick, MD, USA) and were cared according to the regulations and policy for the care and use of laboratory animals published by the Seoul National University. Animals were maintained in the laboratory animal facility under a 12h light/dark cycle. Temperature was controlled at 23°C±2°C and 50%±10% humidity. For aerosol gene delivery, mice were exposed to the aerosol in a nose-only exposure chamber following previously established methods. K-ras^LA1^ mice were randomly divided into three groups of control, scrambled siRNA, and siOPA1.

### Gel retardation assay

Gel retardation assay was performed by a common gel electrophoresis. After complexation of0.1μg of pGL3 plasmid with FPS, the siOPA1 and pGL3 gene condensation ability of the FPS co-polymer was confirmed by gel electrophoretic technique according to various N/P (amine group of polymer / phosphate of gene) ratio group from 1 to 20. The complexation of FPS / siRNA (1ug) wasdiluted in RNase-free DEPC-treated water and then incubated at room temperature(RT) for 30min. Final volume of the solution was 15μL including agarose gel loading dye(2μl) and ethidium bromide(EtBr) (1μL / tube). To evaluate the release ability and complexation, the FPS/siRNA complexes were loaded onto 2% agarose gels and electrophoresed at 100V for 30min with Tris/Borate/EDTA (TBE) buffer.

### The protection and release assay of siRNA

siRNA protection in a RNase A environment and release of siRNA from polymer complexes could be investigated by a gel electrophoresis process. FPS/ siRNA(control siRNA) complexes and naked siRNA (0.5ug) were separately incubated for 30min at RT. 0.1U RNase A (0.2ul) was added to siRNA and FPS/siRNA complexes for 30min at 37°C prior to addition of glycerol. Each group was loaded into 2% agarose gel in 1X TBE buffer.

### *In-vitro* cell cytotoxicity with pGL3 gene and siOPA1

Cell viability of three different cell lines (A549, HepG2 and 293T) was measured with MTT assay. Cells were seeded at a density of 1 x 10^5^ cells/well in a 24-well plate and grown to 70% for 24H. Then incubated with various ratio of PEI, PSMT and FPS / pGL3 gene (0.5μg/ml) complexes in the 500μl media without serum for 24h. On the other hand, polymers/siOPA1 complexes incubated with polymer/siOPA1 complxes in the 500μl media without serum for 48H and 72H. After incubation, MTT reagent (0.5mg/ml) was added to the relevant well in the culture medium for 4H for the reaction to occur. After 4H, media was aspirated carefully and DMSO was added to each well to dissolve purple formazan.After the addition of DMSO, solution was transferred to 96-well plates and the optical density was measured at 535-540nm using a VersaMax tunable micro-plate reader (Sunnyvale, USA). The results were converted into % viability by using the absorbance from untreated wells as a reference, and expressing the absorbance obtained from the treatment groups as a percentage of the reference value.

### *In-vitro* transfection efficiency with pGL3 gene luciferase siRNA

Transfection efficiency (*in-vitro*) in three different cell lines (A549, HepG2 and 293T) was measured by luciferase assay. Cells were seeded at density of 1 x 10^5^ cells/well and grown to 70~80% confluence in 24-well plates. Luciferase positive control siRNA were complexed with FPS, PSMT and PEI at various ratio. Serum-free media containing complexes were added to each well and incubated for 4H. Media were replaced with the serum and incubated for 24H. Used siRNA were incubated for 48h. After aspirating the media, 1x passive lysis buffer 100μl was added to each well. Luciferase assay was performed according to the manufacturer’s instruction (luciferase assay system protocol-Promega). Luciferase activity was detected using luminometer (Infinite®200 PRO, TECAN, Switzerland) to measure relative light units (RLU), which were normalized via measured total protein by a BCA protein assay.

### Competition assay

Competition assay was performed to confirm the lung cancer targeting efficacy of FPS / siRNA complexes at an N/P ratio 20. A549 cells were seeded at density of 1 x 10^5^/well and incubated for 18-20H to obtain 80–90% confluence in 24-well plate. Before transfection of FPS / siRNA complexes, folate as a competitor was prepared by diluting in serum-free media with various concentrations (0mM, 10mM, 20mM, 30mM) and treated to each well. Solution was then incubated for 30min. As mentionedearlier, the transfection of FPS /siRNA complexes was performed, and luciferase activity was confirmed by same method with *in-vitro* cell transfection process.

### Effect of cellular uptake inhibitors on transfection efficiency

To verify endocytosis pathway mechanism of FPS/siRNA complexes, A549 cells were seeded at 1 x 10^5^cells/well and grown to 70~80% confluence in 24-well plate. Chloro-promazine, Genistein and ortmannin, known as cellular uptake inhibitor, were prepared by diluting in DMSO, then mixed with serum free media at various concentrations and treated to the cells. Solution was incubated for 1H at 37°C in a humidified atmosphere of 5% CO2. After the incubation PEI/siRNA, PSMT/siRNA and FPS/siRNA complexes at N/P 10 were treated and incubated with inhibitors for 4H. Effects of cellular uptake inhibitors were investigated by measuring luciferase activity in triplicate.

### Effect of bafilomycin A1 on transfection efficiency

A549 cells were seeded at density of 1 x 10^5^ cells/well and incubated for 18-20H. When cells reached 70~80%, bafilomycin A1 (200nM, 250μl/well), an inhibitor of vacuolar-type H^+^-ATPase (V-ATPase), was treated to the cells for 15min. Cells were then transfected with PEI/siRNA, PSMT/siRNA and FPS/siRNA complexes at N/P 20. Luciferase activity was measured as described above.

### Western blot of OPA1 protein

Lungs were homogenized, and protein concentrations were measured with a BCA protein assay kit (Thermo Fisher Scientific, Waltham, MA, USA). An equal amount of protein (25 mg) was separated in a SDS gel. Appropriate primary antibodies were purchased from Actin-HRP (Santa Cruz Biotechnology, Santa Cruz, CA, USA) and OPA1 (BD Biosciences, San Jose, CA, USA). Secondary antibodies conjugated with horseradish peroxidase (Life Technologies, Grand Island, NY, USA) were applied according to the manufacturer’s protocols. Quantification of the proteins were obtained with the CS analyzer 3.0 program (ATTO, Tokyo, Japan).

The OPA1 protein(50ug) were separated on SDS-PAGE and transferred to the nitrocellulose membranes using a wet transfer method on 12V for 1H. The membranes were pre-blocked for 1H in Tris-buffered saline (TBS) + Tween 20 solution containing 5% skimmed milk overnight at 4°C with agitation. Subsequently, solutions were incubated with either base-1 IgG (rabbit polyclonal antibody) or GAPDH IgG (rabbit polyclonal antivbody) overnight at 4°C. After washing by TBS+Tween 20 for 15min, goat anti-rabbit IgG-HRP was treated and incubated for 1H with agitation. The bands of interest were detected with CCD cameral gel documentation system(chemidoc; Bio-rad, USA).

### *In-vitro* apoptosis of OPA1 protein

The Annexin V-FITC Apoptosis Detection Kit I (Koma Biotech, Seoul, Korea) was used to detect apoptosis by flow cytometry. After treatment of Polymer/siRNA complexes, fluorescence intensity in the cells was measured using FACS AriaII flow cytometer (BD, Franklin Lakes, NJ, USA). Early-stage apoptotic cells were annexin V positive, and PI negative. Late-stage apoptotic cells were annexin V positive, and PI positive. Necrotic cells were annexin V negative, and PI positive.

### *In-vivo* aerosol delivery

All animals used in this study were maintained under animal protocols of Seoul National University guidelines and the study was approved by the Seoul National University Institutional Animal Care and Use Committee (SNUIACUC, Seoul, Korea). Breeding K-ras^LA1^ mice, which are an accepted model of human non-small cell lung cancer, were obtained from Human Cancer Consortium-National Cancer Institute (Frederick, MD) and kept in the laboratory animal facility with temperature and relative humidity maintained at 23 ± 2°C and 40 ± 20%, respectively, under a 12-hour light/dark cycle. Experiments were performed on 8-week-old female K-ras^LA1^ mice (n = 6 per group). The control group was untreated. The other two groups were exposed to an aerosol containing polymer with siSCR and siOPA1. The K-ras^LA1^ mice exposed to siRNA complexes were placed in a nose-only exposure chamber twice a week for 4weeks.

Scrambled siRNA (sense: 5’-*CGUACGCGGAAUACUUCGAUU*-3’; antisense: 5’-*UCGAAGUAUUCCGCGUACGUU*-3’) and mouse siOPA1 (sense: 5’-*GAG GAUAGCUUGAGGGUUA*-3’; antisense: 5’-*UAACCCUCAAGCUAUCCUC*-3’) were used for experiment. At the end of the study, mice were sacrificed and the lungs were carefully collected. During necropsy, the neoplastic lesions of lung surfaces were carefully counted under a microscope. Simultaneously, the lungs were fixed in 10% formalin for histopathological examination. After experiments, remaining lungs were stored at −70°C for further analysis.

### Histopathology

The lung tissues were collected and fixed in 10% formalin. After paraffin embedding, tissue sections were cut at a thickness of 4 μm and transferred to a slide using microtome blade (Leica Biosystems, Nussloch, Germany). For histological analysis, the tissue sections were routinely processed and stained with hematoxylin and eosin. Sections were deparaffinized, rehydrated, antigens were retrieved, and endogenous peroxidase was quenched for immunohistochemistry. Primary and secondary antibodies and 3, 3’-diaminobenzidine (DAB) were applied accordingly (Life Technologies).

All of H&E slides were scanned using ScanScope AT (Aperio Technologies, Vista, CA) to measure the thicknesses of intestinal epithelium.

### Statistical analysis

Statistical analysis was performed by GraphPadPrism 5 (GraphPad Software, San Diego, CA). Student’s *t*-test was used and presented as the mean ±SEM. The *p*-values of less than 0.05 were considered statistically significant. (* p < 0.1 was considered significant and ** p < 0.05 and *** p < 0.01 were considered highly significant compared with the corresponding control).

## Results and discussion

### Synthesis and characterization of PSMT and FPS

**Fig 1:**
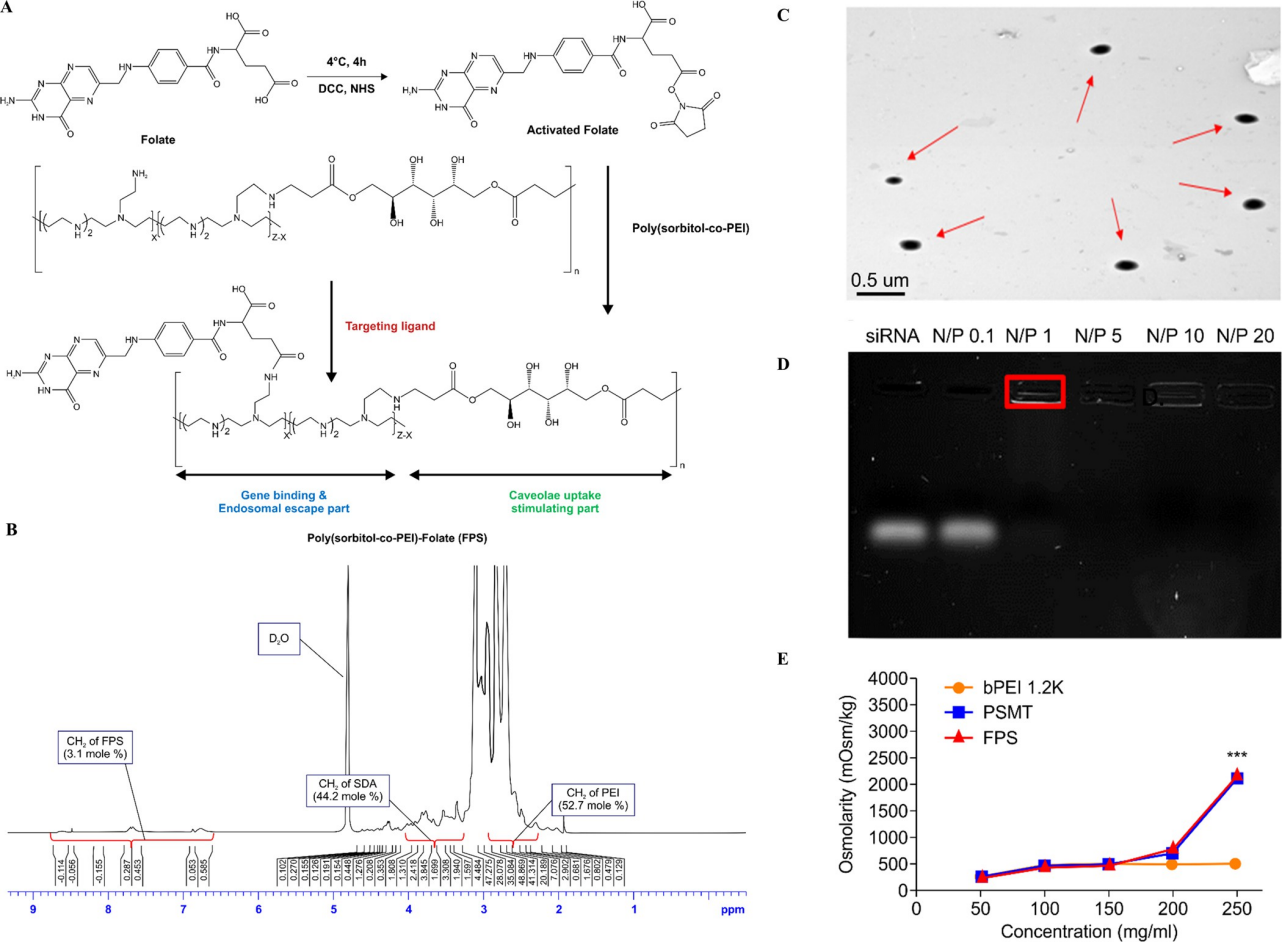
Synthesis and characterization of FPS. (A) Schematic illustration of FPS synthesis. (B) ^1^ H NMR spectra PEI, SDA and FA in FPS. (C) energy-filtering transmission electro-microscopy (EF-TEM) at N/P ratio 20, (D) Electrophoresis, siRNA complexed with FPS at N/P ratio 0.1, 1, 5,10 and 20, E; Osmolarity of PEI 1.2 kDa, PSMT and FPS measured by osmometer.

Sorbitol diacrylate (SDA) was prepared by reacting sorbitol with acryl chloride in a molar ratio of 1:2 with presence of pyridine at 4°C for 24H. Diacrylate can be introduced only to positions 1 and 6 of sorbitol’s hydroxyl groups, because these positions on primary alcohols are more reactive than the hydroxyl groups of secondary alcohols. PSMT was prepared from SDA and LMW PEI (MW:1.2kDa) in anhydrous DMF at 80°C for 24h. Finally, an ester linkage was formed via the reaction of the amide group of SDA and the amine group of LMW PEI [6]. (S1 Fig) FA was activated with DCC and NHS solutions at 4°C. After activation, FA was directly conjugated with PSMT. The reaction scheme and functional parts of FPS polymers are described in Fig 1A. The synthesis of FPS can be confirmed with an ^1^H NMR assessment of the peaks of the vinyl groups in the primary alcohol position of the SDA(δ:3.1–4.1)-backbone and PEI(δ:2.4–2.8) in FPS. (Fig 1B) The compositions of PEI, SDA, and FA were estimated at 52.7%, 44.2% and 3.1% mol, respectively, via ^1^H NMR analysis. For accuracy, the molecular weight of FPS was measured by GPC at a value of 10,951 Da, as shown in Fig 1C and S1 Table.

### Physicochemical characteristics as gene transporter

In a gene delivery system, carriers play an essential role in determining the particle size and surface charge distribution and toxicity [11]. FPS/siRNA complexes, ranged from 145.2 to 192.4nm are much smaller than FPS/DNA polymers (S2 Table), implying that they are more suitable for gene delivery. The particle sizes of FPS/siRNA and FPS/DNA complexes at an N/P ratio of 20 monitored by energy-filtering transmission electro-microscopy (EF-TEM) are significantly similar to those measured by dynamic light scattering. EF-TEM also revealed that the particles are compact and spherical in shape (Figs 1C and S1B).

Additionally, the particle sizes of FPS/siRNA complexes at N/P ratio 20 were monitored by TEM in order to confirm the values for polymer DLS, the zeta potential of FPS/siRNA complexes ranged from +6.6 to +9.4 (S3 Table), which is significantly lower than the range for PEI/siRNA complexes. This means that the positive charge on the FPS/siRNA polymer was neutralized by the hydroxyl group of SDA.

Gel retardation assay conducted for evaluation the electrostatic interaction and condensation with cationic FPS and negatively charged DNA. As a result of electrophoresis, confirmed no band by perfect neutralization and condensation at N/P ratio 1 (S1C Fig), it means that electrostatic self-assembly of FPS/DNA complexes indicate perfect complexation.

Additionally, a gel retardation assay confirmed siRNA complexation by FPS. As shown in Fig 1D, no siRNA was observed with neutralization and condensation at an N/P ratio of 1. Also, an siRNA protection and release assay was carried out under an RNase environment to determine whether siRNA complexation with FPS would be protected from enzymatic degradation. Compared with siRNA directly exposed to RNase A, siRNA connected to FPS was unchanged, indicating that genetic cargo can be safely protected and easily released from FPS complexes. (S2 Fig) The osmolarity of FPS is 2.4- and 5.7-fold higher than bPEI 1.2kDa at concentrations of 50 and 250 mg/ml, respectively, which is similar to the proven osmolarity value for PSMT (Fig 1E).

### *In-vitro* cytotoxicity of FPS/siRNA complexes

**Fig 2:**
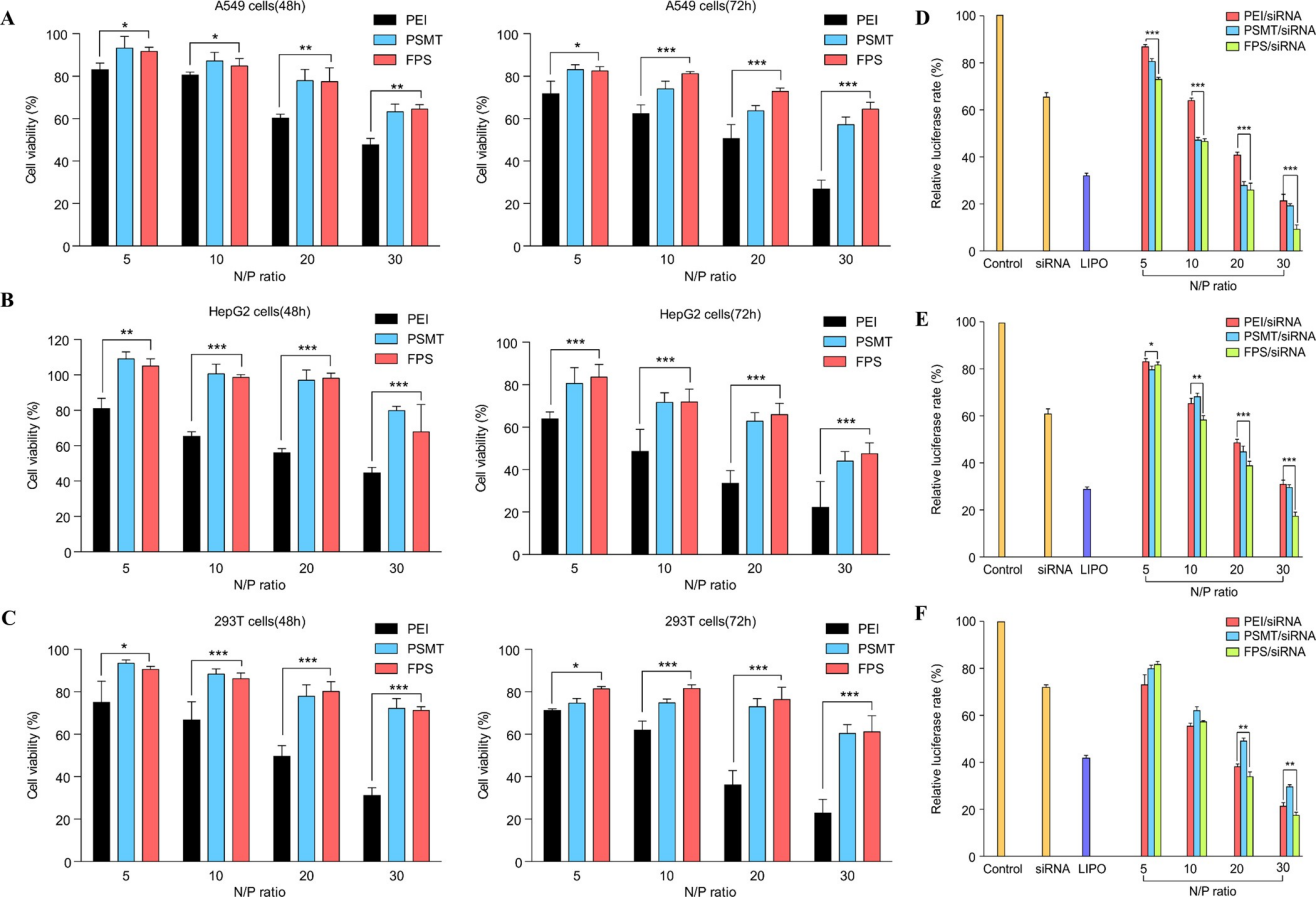
Cytotoxicity of PEI/siRNA complexes. PSMT/siRNA complexes, and FPS/siRNA complexes was measured by MTT assay in A; A549 cells, B; HepG2 cells, and C; 293T cells after 48h and 72h. [*n* = 5, one-way ANOVA, * *p* < 0.1; ** *p* < 0.05; *** *p* < 0.01, compared to that of FPS/siRNA complexes with control (PEI)]. D-F; PEI, PSMT, FPS and Lipofectamine 2000 were complexed with luciferase siRNA at various N/P ratios and transfected in (D) A549 cells, (E) HepG2 cells, and (F) 293T cells without serum. Chemo-luminescence was measured 48h after transfection and normalized with the amount of protein. [*n* = 5, one-way ANOVA; * *p* < 0.1; ** *p* < 0.05; *** *p* < 0.01; compared to that of FPS/siRNA complexes and control groups].

Due to their obstacles in clinical application, non-viral vectors have to meet safety requirements to be used for gene delivery. In this regard, an *in-vitro* cytotoxicity study was conducted using A549, HepG2, and 293T cell lines using MTT assay. As mentioned above, low molecular weight (LMW) PEI was used instead of high molecular weight (HMW) PEI, because some studies indicated that HMW PEI/gene complexes can induce cellular dysfunction due to a high density of the PEI amine group [12]. The connection between the amine group of LMW PEI and the amide group of SDA by ester linkage and the negative charge of SDA’s hydroxyl group both help to quench PEI’s positive charges, which brings advantages including improved cell viability. Cells were treated with various N/P ratios (5, 10, 20, and 30) of FPS/siRNA complexes, PEI/siRNA, and PSMT/siRNA polymers for 48h and 72h. These results show that the FPS/siRNA complexes were less cytotoxic compared with PEI (25 KDa) in broad N/P ratios, both after treatment for 48h and 72h, implying that the negative charge on the 4-hydroxyl groups of the poly-sorbitol part of FPS may be responsible for the neutralization of the positive charges on the PEI part of FPS, potentially bringing a multifunctional effect that contributes to the improvement of cell viability (Figs 2A–2C and S3).

### *In-vitro* transfection efficiency of FPS/siRNA complexes

As mentioned above, non-viral vectors for gene delivery have limitations, such as insufficient transfection efficiency compared with viral vectors. To confirm the *in-vitro* transfection ability of FPS, pGL3 luciferase reporter plasmids were delivered using PEI, PSMT, FPS and Lipofectamine 2000 in identical cell lines with an MTT assay. To analyze the silencing efficacy of FPS as a shuffling control for siRNA, FPS/siLuciferase complexes were transfected in A549 cells, HepG2 cells, and 293T cells for 48h and analyzed via the bioluminescence method. As shown in Fig 2D–2F, a greater Luciferase silencing efficiency was observed as the N/P ratio of nanoparticles increased. In the case of A549 cells, targeting reporter protein Luciferase using anti-Luciferase siRNA with FPS and PSMT carriers showed efficient silencing compared to PEI25K and Lipofectamine2000 with luciferase siRNA in a broad range of N/P ratios (5–30). An N/P ratio of 20 for FPS/luciferase siRNA complexes showed 1.8-fold higher silencing efficacy than that of PEI25K. Additionally, silencing efficacy at an N/P ratio of 30 for FPS/luciferase siRNA complexes was 3-fold and 2-fold higher than that of Lipofectamine2000 and PEI 25k, respectively. At all N/P ratios of polymer/siRNA complexes, FPS displayed superior transfer efficiency over the PEI25K carrier. The results of consistent trends were also confirmed in FPS/DNA complexes (S4 Fig). These results indicate FPS as a potential siRNA transfer carrier, and also highlight the importance of a proper N/P ratio in down-regulated target proteins.

### Mechanism of gene delivery by FPS

**Fig 3:**
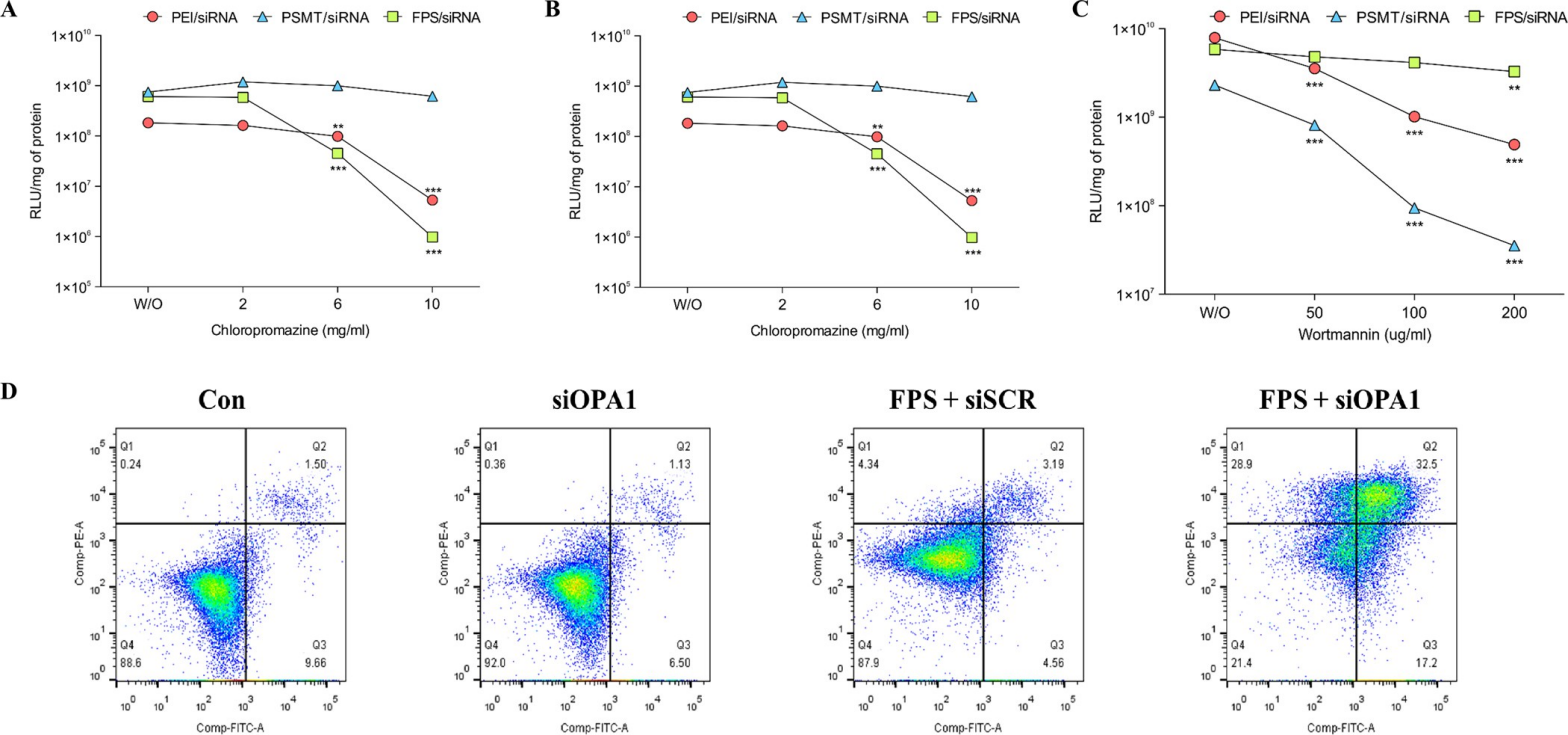
Endocytosis inhibitors on transfection efficiency of FPS/siRNA complexes and measurement of induced apoptosis by siOPA1. (A)-(C) Endocytosis inhibitors on transfection efficiency of FPS/siRNA complexes. Cells pre-incubated with various amount of chloropromazine, genistein, and wortmannin were transfected with PEI, PSMT, and FPS/siRNA complexes (N/P ratio 20) and luciferase assay was conducted. (n = 3, one-way ANOVA; * p < 0.1; ** p < 0.05; *** p < 0.01, compared to that of control without inhibitor), (D) Measurement of induced apoptosis by siOPA1, All groups combined annexin-V and viability dyes PI in A549 cells.

It became evident that modification of polymers with ligands to fit internalizable receptors results in the achievement of cell specificity and receptor-mediated internalization into the target cell [13]. To reveal pathways of FPS/siRNA complex penetration, experiments with three different types of inhibitors of different endocytosis pathways were carried out. The transfection efficiencies of FPS, PEI, and PSMT/siRNA complexes were investigated in the presence of Chloro-promazine (CH) as an inhibitor of clathrin endocytosis, Genistein (GE) as an inhibitor of caveolae endocytosis, and Wortmannin (WO) as an inhibitor of fluid-phase endocytosis [14]. As shown in Fig 3A–3C, treatment with increasing concentration of CH and GE drastically decreased the transfection level of FPS/siRNA complexes compared with PEI/siRNA complexes, which strongly suggests that both caveolae endocytosis and clathrin endocytosis largely contributed to the entry of FPS/siRNA polyplexes into lung cancer cells.

In contrast, treatment with WO shows a small decrease in the permeability of FPS/siRNA complexes compared with that of PEI/siRNA, implying that FPS/siRNA complexes are less dependent on the fluid-phase endocytosis pathway than other pathways. These results suggest the potential application of the FPS polymer as an effective gene carrier, due to its ability to protect the lysosomal degradation fate of internalization via selective internalization of the hyperosmotic FPS polymer by caveolae endocytosis.

### Competition assay of FPS/siRNA complexes

To investigate the uptake mechanism of FPS through interactions between folic acid parts in the FPS and folate receptors in lung cancer cells, a competition assay was conducted by adding broad concentrations of free folate (0,0.01,0.1, and 1mg/ml) as the competitor. Pre-treatment with free folic acids at concentrations of 0.01, 0.1 and 1mg/ml drastically decreased luciferase activity of FPS. This result indicates that cellular uptake of FPS/siRNA complexes is mediated by receptor-mediated endocytosis via interactions between FPS ligands and FPS receptors (S5 Fig).

### Proton sponge effect

After cellular internalization, intracellular barriers, such as safe releasing of the vector into the cytoplasm from the endosome, can be major bottlenecks for the efficiency of a gene delivery system. Therefore, PEI has drawn the most attention as a potential cationic non-viral vector due to its specific ability, known as the “proton sponge effect" [15]. This hypothesis suggests that the buffering capacity of PEI during the acidification of the endosome results in the entrance of chloride anions to maintain electro-neutrality, which finally leads to the osmotic swelling and rupture of endosomes prior to fusion with lysosomes [16].

To verify the proton sponge effect of polymers, we used 200nM of bafilomycin A1 as specific vaculoar type H+ ATPase inhibitor. After pre-treatment bafilomycin A1, transfected the FPS/siRNA complexes at an N/P ratio 20 in the A549 cells. As a result, bafilomycin A1 significantly reduced the transfection efficiency of FPS/siRNA polyplexes and PEI/siRNA complexes by 25 fold and 40 folds. This result strongly suggests that the proton sponge effect contributes to the endosomal escape of FPS/siRNA complexes (S6 Fig).

### *In-vitro* apoptosis of OPA1 protein

Silencing OPA1 with siRNA in A549 cells induces apoptosis and necrosis. A549 cells, which had been treated with FPS/siOPA1, FPS/siSCR and siOPA1 for 24h and then combined with annexin V and PI, were stained to allow for distinctions to be made between living cells, early apoptotic cells, late apoptotic cells, and apoptotic necrotic cells. One day post-treatment, approximately 50% of A549 cells showed apoptosis with FPS/siOPA1 treatment and 30% of cells showed necrosis. Interestingly, compared with the results of FPS/siOPA1, less than 20% of A549 cells displayed either apoptosis or necrosis when treated with FPS/siSCR or siOPA1 (Figs 3D and S7). These results present FPS as a potential siRNA delivery vehicle, and also highlight siOPA1 as a cargo that can potentially be used to induce apoptosis.

### Anti-cancer effect of FPS/siOPA1 complexes

**Fig 4:**
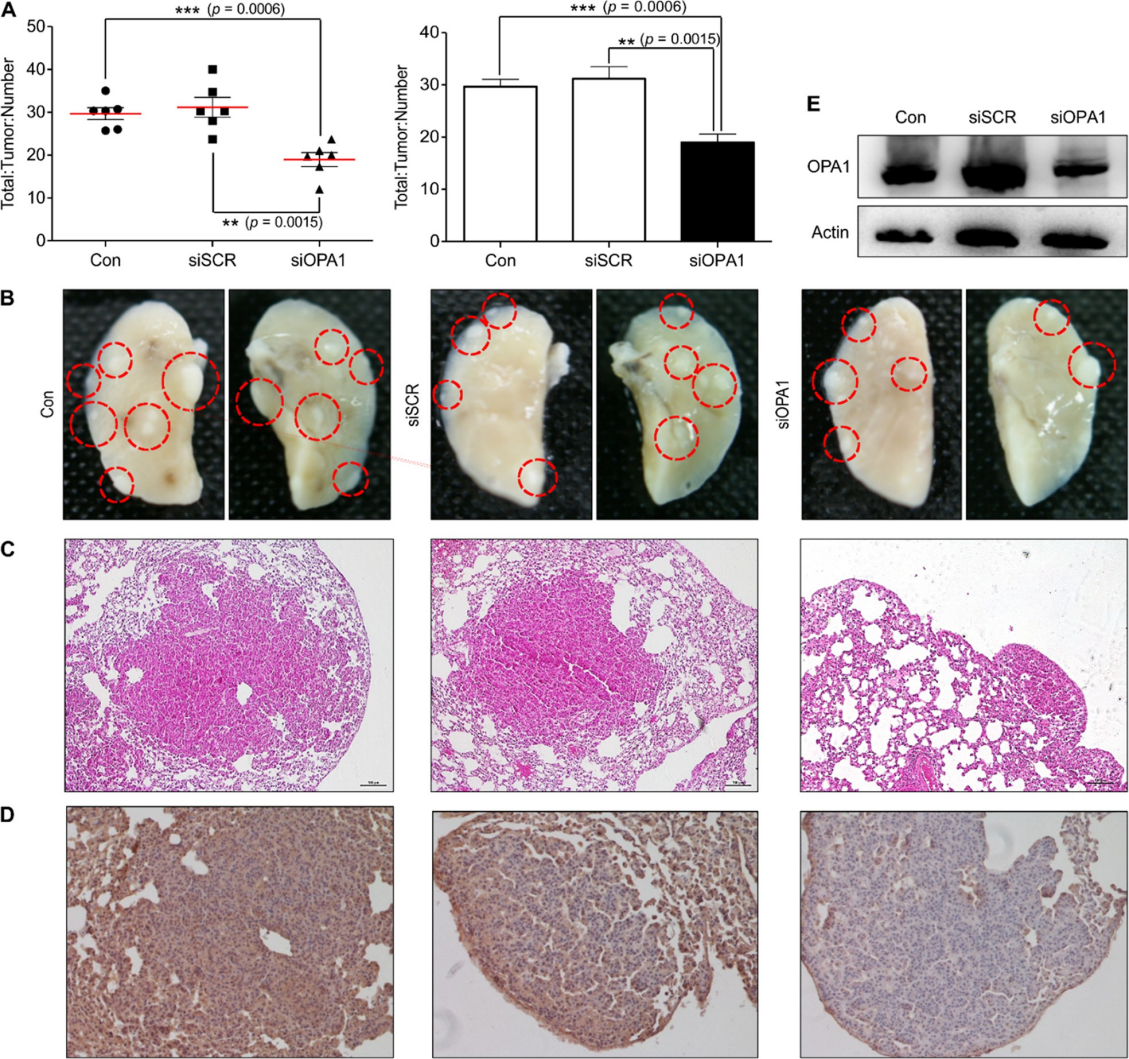
Therapeutic efficiency of FPS as aerosol gene delivery carrier in lung tumor bearing K-rasLA1 mice. Aerosol delivery FPS/siOPA1 significantly inhibited lung tumor numbers; (A) A decrease in the number and size of tumor nodules were found during gross morphological observations. (B) The total number of tumors decreased significantly in the FPS/siOPA1-delivered group. (red circle represents tumor tissues). (C) The control group and FPS/siSCR group showed severe thickening of alveolar septa with invasive growth and loss of normal lung structure, whereas the FPS/siOPA1 group showed a smaller tumorigenic region. (D) Immunohistochemistry reconfirmed downregulation of OPA1 in tumor-bearing lungs of FPS/siOPA1-delivered mice. (E) Western blot analysis of OPA1 protein expression in the lungs and bands-of-interest were further analyzed by densitometer, confirmed down-regulation of OPA1.

Based on *in-vitro* results, aerosol delivery of a gene is a possible method for targeting lung cancer cells in *in-vivo* experiments. The anatomical structure and location of the lungs allow instant access for genetic cargos transported by inhalation, with a high and safe loading efficiency [17]. To evaluate whether the knockdown of OPA1 has potential as an anti-cancer therapy through apoptotic cell death, exposure to FPS/siOPA1 complexes in K-ras^LA1^ lung cancer was modeled in mice [18]. As shown in Fig 4, significant anticancer effects of FPS/siOPA1 complexes, delivered to the lungs via aerosol inhalation, were observed without toxicity. They promoted high transfection efficiency with significantly decreased tumor formation. These results lend the possibility that the FPS co-polymer could be a good siRNA carrier in aerosol-administered lung cancer gene therapy. These results also suggest that the knockdown of OPA1 causes suppressed tumor progression in lung adenocarcinoma.

## Discussion

A new non-viral vector FPS composed of PEI, poly-sorbitol and folate was designed as an advanced gene delivery system. The FPS proved to possess various ideal features: (1) the ability to be synthesized easily within a relatively short amount of time, (2) a suitable particle size for gene delivery, (3) significantly low cytotoxicity, which can be achieved by implementing low MW PEI ester-linkage with the amide groups of SDA, (4) ideal transfection efficiency due to the synergistic effect of backbones in FPS, and (5) the rapid endosomal escape of genes causes gene degradation due to the proton sponge effect of PEI. Most importantly, FPS was able to be loaded with siRNA such as si-Luciferase and siOPA efficiently. In both *in-vitro* and *in-vivo* cases, western blot analysis indicated that FPS/siOPA1 had a higher silencing effect on OPA1 gene expression compared with other nanoparticles, and this makes FPS a potential therapeutic agent for treating lung cancer. Our FPS/siOPA1 delivery system has biocompatibility, low cytotoxicity, and a biodegradable carrier. The system includes effective antitumor functions, and with all of the above features combined, the FPS/siOPA1 gene delivery system provides a novel and versatile approach that can offer novel insights in the biomedical field.

## Supporting information

S1 Fig Synthesis and characterization of FPS. (TIF)

S2 Fig siRNA protection assay. (TIF)

S3 Fig Cytotoxicity of FPS/DNA and PEI/DNA complexes was measured by MTT assay method in (A) A549 cells, (B) HepG2 cells, and (C) 293T cells. (TIF)

S4 Fig In vitro transfection efficiency of FPS/DNA complexes. PEI, PSMT, FPS and Lipofectamine 2000 were complexed with pGL3 gene at various N/P ratios and transfected in (A) A549 cells, (B) HepG2 cells (C) 293T cells without serum.
(TIF)

S5 Fig Competition assay of FPS/luciferase siRNA complxes on A549cells. (TIF)

S6 Fig Effect of Bafilomycin A1 of FPS/siRNA complexes in A549 cells. (TIF)

S7 Fig Measurement of induced apoptosis by siOPA1. (TIF)

S1 Table Molecular weight of PSMT, FPS measured using GPC. (XLSX)

S2 Table Particle size(nm) of PEI/DNA, FPS/DNA complexes and PEI/siRNA, FPS/siRNA complexes. (XLSX)

S3 Table Zeta potential of PEI and FPS/DNA complexes and PEI/siRNA, FPS/siRNA complexes. (XLSX)

S1 File (PDF)
