# Correction: Efficient gene transfection to lung cancer cells via Folate-PEI-Sorbitol gene transporter

**DOI:** 10.1371/journal.pone.0306665

**Published:** 2024-07-01

**Authors:** The PLOS ONE Staff

The affiliation for the sixth author is incorrect. Ah Young Lee is not affiliated with #4 but with #3: Laboratory of Toxicology, Research Institute for Veterinary Science and College of Veterinary Medicine, Seoul National University, Seoul, Republic of Korea.

The publisher apologizes for the error.
