# Supplementary figures and images for: Efficient gene transfection to lung cancer cells via Folate-PEI-Sorbitol gene transporter
Source: PLoS One. 2022 May 4;17(5):e0266181. doi: 10.1371/journal.pone.0266181 (PMC9067668; doi:10.1371/journal.pone.0266181)

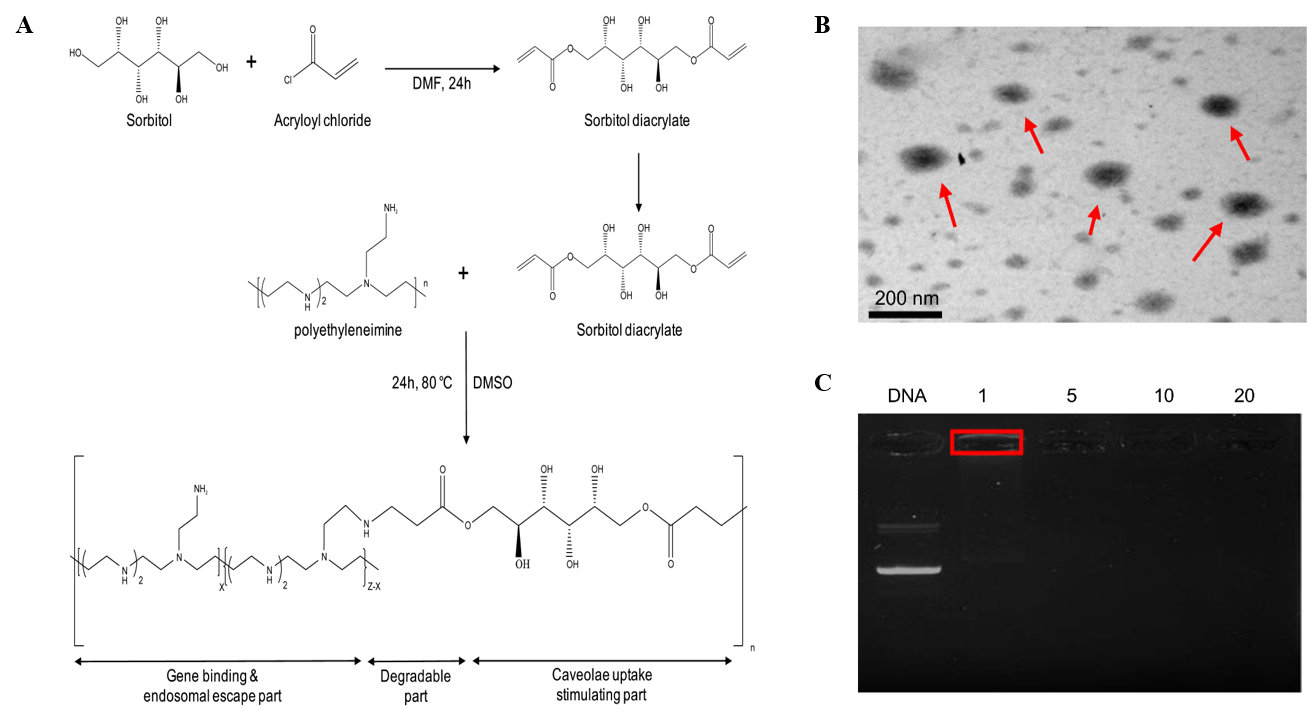

Supplement: S1 Fig — (TIF) [file pone.0266181.s001.tif]

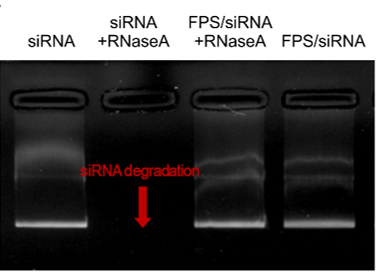

Supplement: S2 Fig — (TIF) [file pone.0266181.s002.tif]

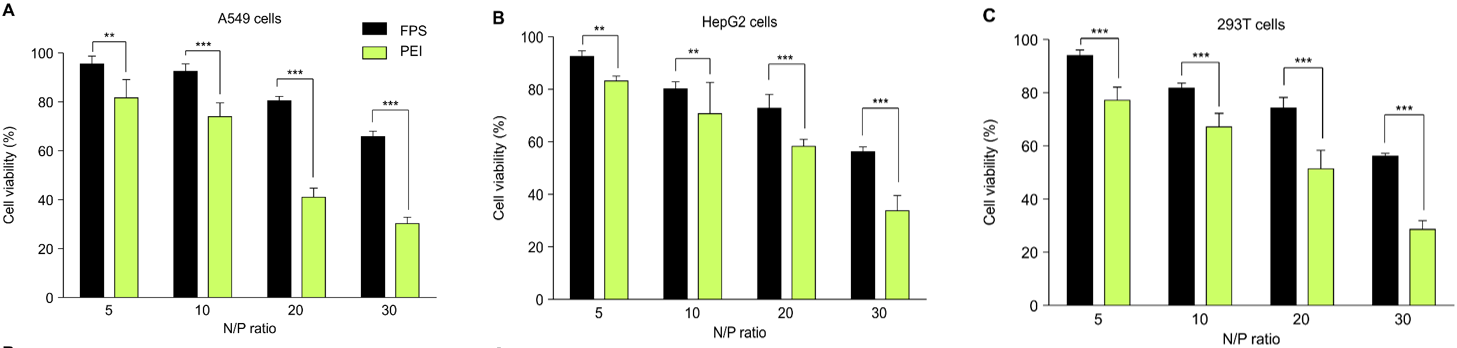

Supplement: S3 Fig — (TIF) [file pone.0266181.s003.tif]

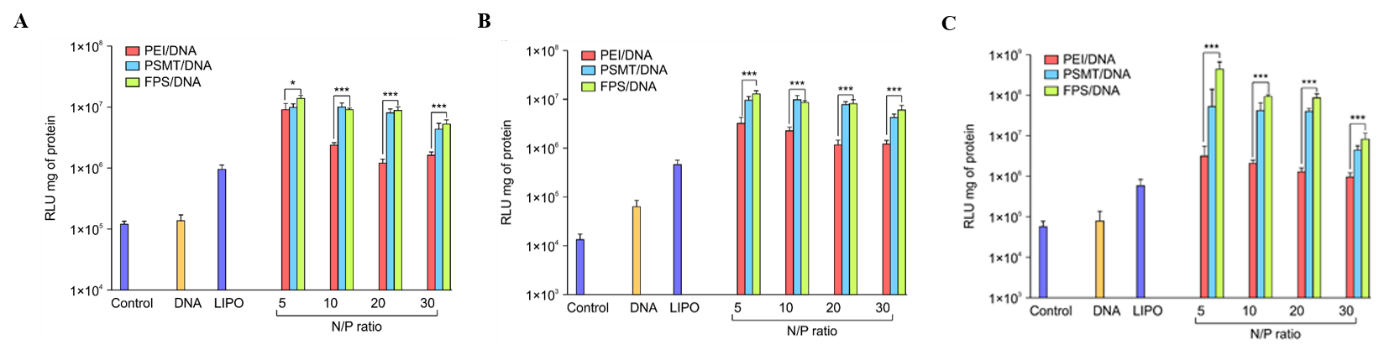

Supplement: S4 Fig — PEI, PSMT, FPS and Lipofectamine 2000 were complexed with pGL3 gene at various N/P ratios and transfected in (A) A549 cells, (B) HepG2 cells (C) 293T cells without serum. (TIF) [file pone.0266181.s004.tif]

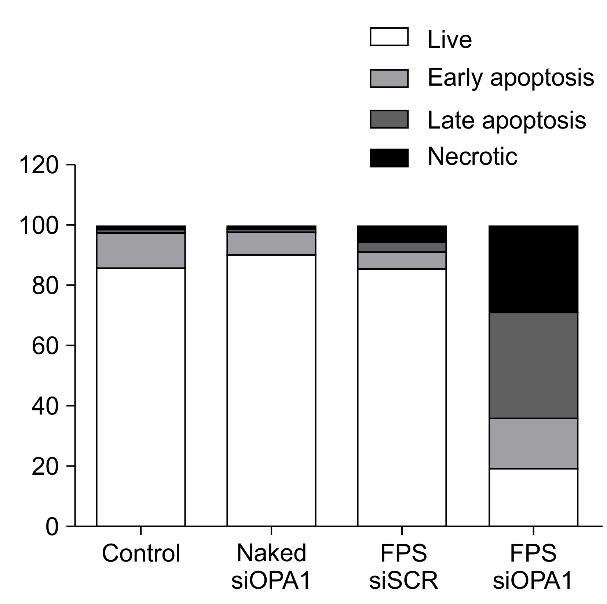

Supplement: S7 Fig — (TIF) [file pone.0266181.s007.tif]
