# Supplementary material for: Efficient gene transfection to lung cancer cells via Folate-PEI-Sorbitol gene transporter
Source: PLoS One. 2022 May 4;17(5):e0266181. doi: 10.1371/journal.pone.0266181 (PMC9067668; doi:10.1371/journal.pone.0266181)
Supplement: S1 File — (PDF) [file pone.0266181.s011.pdf]

Figure 1D. Electrophoresis, siRNA complexed with FPS at N/P ratio 0.1, 1, 5,10 and 20

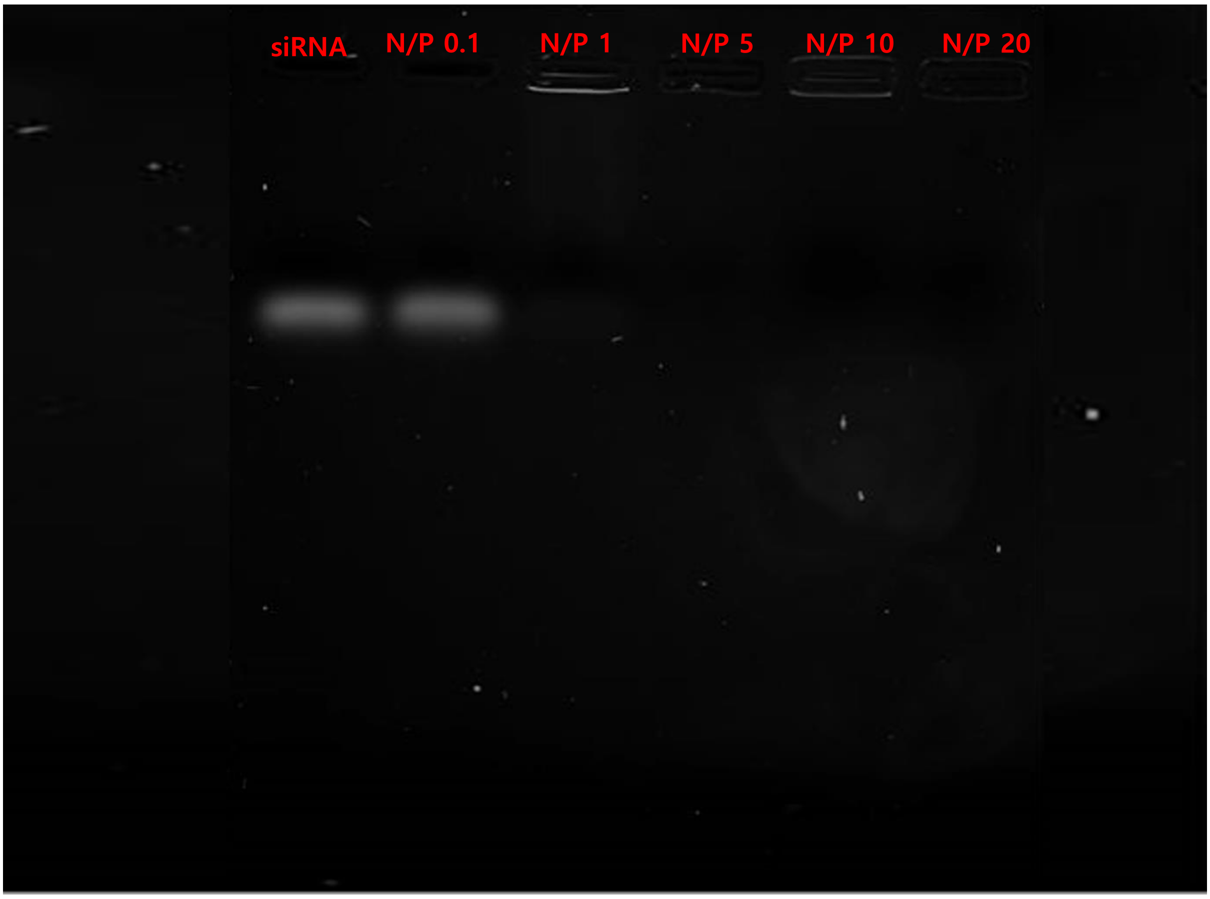

Main figure 1b-image in the paper is the original image. (6 wellss)

Figure 4E. Western blot analysis of OPA1 protein expression in the lungs and bands-of-interest were further analyzed by densitometer, confirmed down-regulation of OPA1.

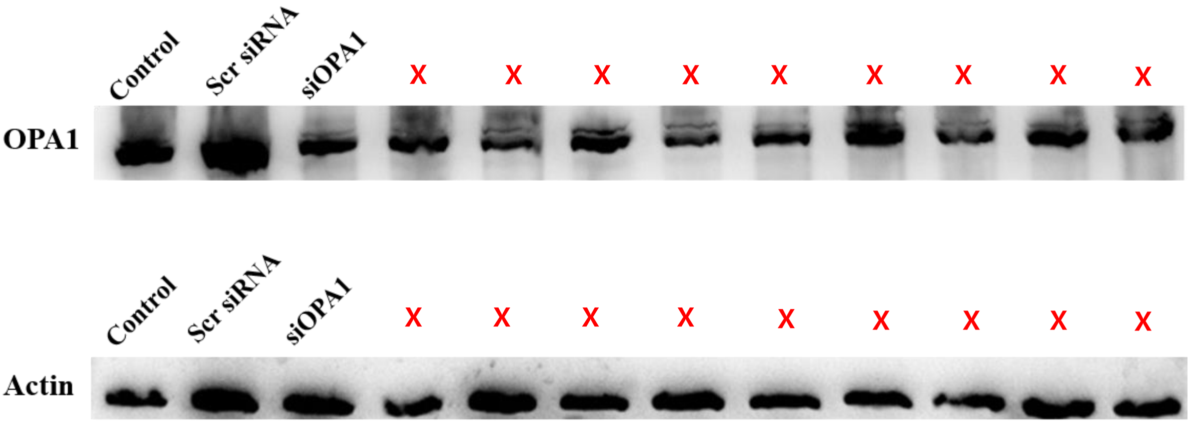

Kimberly-Clark has determined the classification of this information to be "Public"

All lanes not included in the final figure(main figure 4E) should be marked with an "X" above the lane label on the original blot image.

Supplementary Figure 1C. Gel electrophoresis of FPS/DNA complexes at various N/P ratios.

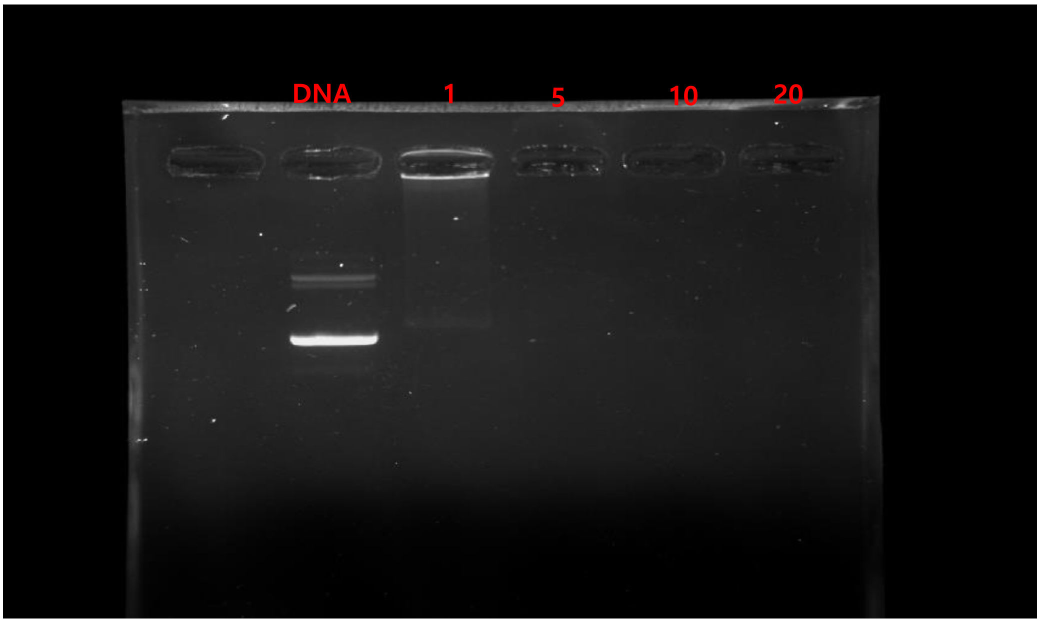

Supplementary Figure 2. siRNA protection assay, siRNA was released from by FPS/siRNA complexes by SDS.

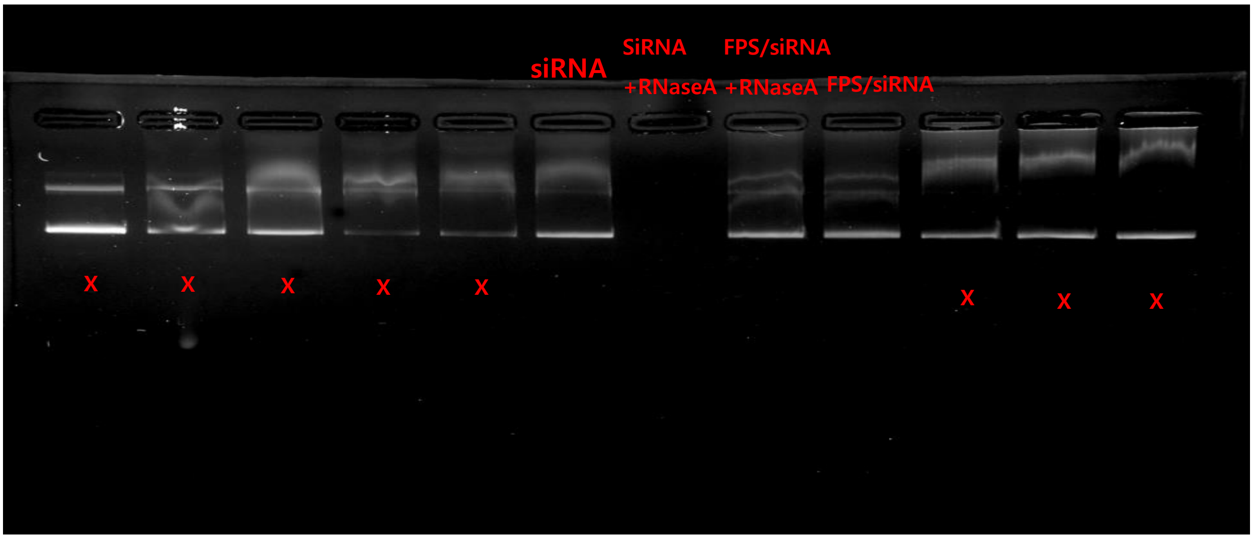

Kimberly-Clark has determined the classification of this information to be "Public"
